# Barriers to mental health help-seeking in veterinary professionals working in Australia and New Zealand: A preliminary cross-sectional analysis

**DOI:** 10.3389/fvets.2022.1051571

**Published:** 2022-11-04

**Authors:** Caitlin Elizabeth Connolly, Kimberley Norris, Sarah Dawkins, Angela Martin

**Affiliations:** ^1^School of Psychological Sciences, University of Tasmania, Hobart, TAS, Australia; ^2^Menzies Institute for Medical Research, University of Tasmania, Hobart, TAS, Australia

**Keywords:** help-seeking, barriers, psychopathology, occupational mental health, veterinary, cultural norms

## Abstract

Despite higher reported rates of mental ill-health than the general population, professionals working in the animal care industry have low reported rates of help-seeking behavior. Potential factors involved in veterinary professionals' reluctance to seek help include stigma toward mental ill-health, practical barriers to accessing supports, and a cultural normalization of symptoms in the industry. This preliminary study sought to explore these factors in a sample of veterinarians, veterinary nurses, and veterinary technicians and examine effects of gender, years' experience, and practice location. A total of 408 veterinary professionals working in Australia and New Zealand completed an online survey between June and December 2021 measuring perceived stigma, practical barriers to mental health help-seeking, perceptions of normalized psychopathology and sickness presenteeism. Results indicated moderate levels of both perceived stigma and barriers to mental health help-seeking. Interestingly, psycho/pathology (e.g., burnout, fatigue, and sickness presenteeism) was perceived to be a normalized aspect of the profession by majority of respondents. Although no effect of gender or geographic location were observed, stage of career did have an effect on findings. Early career veterinary professionals were identified as more vulnerable to perceived stigma and barriers to care. The practical and research implications of the findings are discussed and include the need for mental health to be more centrally incorporated into the veterinary curriculum and professional development. Also discussed is an agenda for future research aimed at improving the mental health and wellbeing of professionals working in the animal care industry.

## Introduction

Professionals employed in the veterinary industry have poorer self-reported mental health than the general population (1, 2), with evidence that veterinary professionals working in Australia and New Zealand commonly experience elevated rates of depression, anxiety, burnout and suicidality (3). Factors identified as contributing to these poor psychological outcomes include excessive workloads, poor work-life balance, and the high levels of emotional labor involved with the profession (4, 5).

In addition to these poor psychological outcomes, rates of mental health help-seeking behaviors are known to be low in this industry (6). Several studies have reported low or zero rates of help-seeking among participants reporting severe psychological distress and a history of suicidal ideation and attempts (7, 8). Recent studies have also reported high rates of sickness presenteeism (i.e., employees working while mentally or physically ill or injured) in the veterinary industry, with professionals citing feelings of guilt/burdening their colleagues with extra work and practical obstacles such as staffing shortages as motivating them to present to work while ill (9). This poses several risks including exacerbation of existing ill-health, development of new symptoms, spreading illness to colleagues and clients, and potentially compromising the quality of client care (9, 10).

Studies have cited industry stigma as central to the low rates of mental health help-seeking by veterinary professionals. Stigma toward psychopathology (i.e., symptoms and conditions of mental ill-health and disorder) has been reported to saturate not only human healthcare (6), but also the animal care industry, including tertiary veterinary education (11). Negative attitudes toward mental ill-health found in cohorts of veterinary students are likely due in-part to the culture of competitiveness and high achievement that exists in education in this field. Common traits noted in veterinary students include perfectionism and a significant fear of failure (6, 12). Individuals studying in the industry may synonymize mental ill-health with an inability to handle the required workload and keep pace with their peers. This attitude fosters reticence to disclosing mental health symptoms as students attempt to keep up high expectations of themselves and expectations they perceive from others. Indeed, self-stigma has been found to be significantly higher in veterinary students than their non-veterinary counterparts (13). When students enter the veterinary workforce, this pattern of behavior can endure and solidify as a fundamental barrier to help-seeking. Evidence of this was found in an interview study of veterinarians with a history of suicidal ideation and attempts (8). Participants cited guilt, shame, and fear of losing employment as reasons for non-disclosure of mental health distress. Experiencing psychopathology in an environment of perceived stigma has been demonstrated to elicit low self-esteem (14), thereby exacerbating existing symptoms and placing an additional barrier to engaging with supports.

In addition to the barrier of stigma in the industry, a second possibility that should be considered is that practical barriers are inhibiting access to supports. Professionals may be willing to engage in help-seeking but constrained by factors such as time, location, and lack of information. Time has been cited as the leading barrier to help-seeking in Australian medical professionals (15). Given that long working hours and heavy workloads have been reported as major stressors in the veterinary occupation (6), it is likely time is also preventing access and meaningful engagement with mental health supports in the veterinary industry. Since the emergence of the COVID-19 pandemic, these hours and workloads have been further extended as pet ownership has reached unprecedented levels (16). These practical barriers may be especially relevant to professionals working in rural and remote communities serving livestock and the agricultural sector. There is significantly less psychological support and information available to populations living in rural and regional areas of Australia compared to metropolitan areas (17). This has been identified as a factor impeding help-seeking in professionals working in human healthcare (15). Thus, beyond the barriers of stigma, it may be that professionals are willing to engage with supports but they do not exist in their area. Although telehealth has risen in uptake since the pandemic, inadequate internet, and insufficient practitioners to meet demand are serving to impede its access in some regional areas (18). There is potential for other demographic factors identified in human healthcare to predict perceptions of barriers to care among veterinary professionals. In a sample of Australian doctors, years' experience was found to significantly predict help-seeking (19). Senior physicians were more likely to seek psychological help than their early career physician colleagues, with the authors citing time as a potential contributor to this difference. It is posited that professionals in the early stages of their career are significantly more time-poor due to study and work commitments. Additionally, professionals in senior roles may possess more autonomy over their schedules and can therefore arrange time to access supports. It is possible this pattern could be observed in animal healthcare due to similar work commitments of early career veterinary professionals. Although it is reported that females experience less stigma toward mental ill-health than males (20), there remain significant barriers that impede females from accessing desired supports. In a sample of female human medical practitioners, key barriers to mental healthcare included inadequate time and concern over consequences for career progression (21).

A third contributor to low help-seeking rates in veterinary professionals to be considered is a normalization of mental health symptoms in the industry. This is a phenomenon also evidenced in human medical care. In a sample of rural and regional Australian doctors, stress was normalized to the extent of being described as “indicative of commitment to the profession or the workplace” [(15) p. 430]. Similar sentiments have been observed in veterinary students (22). If this culture were to persist through veterinary education to the workforce, it would see veterinary professionals' mental health concerns be minimized as “a part of the job”. This could result in low rates of help-seeking behaviors as employees may view their symptoms as the status quo and not a cause for concern. Perceived industry culture of sickness presenteeism for mental ill-health has been identified in cohort of both human (23) and veterinary medical students (22). Sickness presenteeism with symptoms of physical ill-health has been reported in the veterinary industry, with one studying citing two-thirds of participants admitting to attending work despite presenting with symptoms of influenza (9). To encapsulate the breadth of both mental and physical ill-health, the term psycho/pathology will be used hereafter to represent perceptions of normalized mental ill-health (i.e., burnout, fatigue) and physical sickness presenteeism.

### Aim of the present study

A comprehensive understanding of the culture and perceptions of mental health in veterinary practices is essential in improving employee wellbeing. This includes recognizing what is hindering individuals from support-seeking, as well as what may motivate them to engage with supports. Identification of these factors can inform policy change and the development of psychological interventions tailored to the experiences of those studying and working in animal care.

Therefore, the present study aimed to examine barriers to mental health help-seeking, including stigma perceived by veterinary professionals, and provide a preliminary exploration of the normalization of mental health symptoms in the animal care industry. Based on a review of the literature, the authors generated the following research questions for exploration:

**RQ1**: What are veterinary professionals' experiences of perceived stigma and barriers to mental health help-seeking?

**RQ2**: Do groups identified as vulnerable in human healthcare (e.g., early career professionals, professionals employed in rural and remote locations) report higher perceived stigma and barriers to mental health help-seeking than their more experienced and urban-located counterparts?

**RQ3**: To what extent do veterinary professionals agree that psycho/pathology (i.e., burnout, fatigue, sickness presenteeism) is normalized in the industry?

## Materials and methods

An invitation to participate in the study was circulated to 225 veterinary practices and organizations across Australia and New Zealand with the link to an online survey platform. Organizations were identified throughout an online search of currently operating animal care practices and organizations, and randomly selected from those providing contact details to send information about the study. Eligibility criteria to participate included: being over the age of 18 and working in Australia or New Zealand for over 1 year in the role of veterinarian, veterinary nurse, or veterinary technician. Following completion of the questionnaire, participants were given the option to enter a draw to receive a $50 gift card in recognition of their time spent participating. Data was collected from participants between June and December 2021. This study received ethics approval from the Tasmania Social Sciences Human Research Ethics Committee (S0023759).

Demographic information was collected regarding participant gender, occupation, years' experience, type of veterinary practice (i.e., small animal, wildlife, research setting etc.) and workplace location (remote, rural, urban). Data pertaining to occupation type and type of veterinary practice worked in was collected to describe the participant population.

The two subscales encompassing the adapted Perceived Stigma and Barriers to Care for Psychological Treatment Scale (14, 24) were used to measure participants' internalized stigma of mental health symptoms and experience with practical barriers inhibiting their access to care. Both subscales are scored on a 5-point Likert scale (ranging from Strongly disagree to Strongly agree) with higher scores indicating higher perceptions of stigma toward mental ill-health and greater experiences with practical barriers to accessing care. The Stigma subscale comprises 6 items (total score 6–30) and Barriers to Care subscale comprises 5 items (total score 5–25). No systematic scoring information was provided by the scale authors. As such, in the present study scores were trichotomized into low (6–13), moderate (14–22), and high (23–30) for the Stigma subscale, and low (5–11), moderate (12–18), and high (19–25) for the Barriers to Care subscale. Additionally, scores were converted to a format ranging from 1 (low perceived stigma/barriers to care) to 5 (high perceived stigma/barriers to care) to allow for comparison with an external study. The authors report good internal consistency for both subscales (α = 0.82 for the Stigma subscale and α = 0.70 for the Barriers to Care subscale). In the present study, Cronbach's alphas were 0.85 and 0.68 for the Stigma subscale and Barriers to Care subscale respectively.

Industry normalization of psycho/pathology (i.e., burnout, fatigue and sickness presenteeism) was measured by asking participants to indicate the extent to which they agree with three statements (“Fatigue is inevitable in my role”, “Experiencing burnout is considered a normal part of the job”, “I feel pressured to go to work even when I am sick”) on a 5-point Likert scale (ranging from 1 = Strongly disagree to 5 = Strongly agree).

Data was analyzed using IBM SPSS v.27. Frequencies were tabulated for demographic variables and descriptive statistics and correlations were collated for continuous variables. One-way ANOVAs with Tukey *post hoc* comparisons were performed on the two subscales of the Perceived Stigma and Barriers to Care for Psychological Treatment Scale to examine effects of gender, years' experience and practice location. Data collected from the Barriers to Care subscale was further analyzed to determine the barriers most reported by participants by tabulating response frequency. Responses of Agree and Strongly agree were combined to reflect participants had been impacted by that barrier.

Participant experience with normalized burnout, fatigue, and sickness presenteeism was analyzed by examining response frequency. Responses of Agree and Strongly agree were collapsed and percentages tabulated to indicate agreement with each statement. Scores were also summed to indicate total perceived normalization of psycho/pathology with possible scores ranging from 3–15.

## Results

### Demographics

A total of 409 veterinarians, veterinary nurses and veterinary technicians from Australia and New Zealand completed the survey. Data from one participant was excluded from analysis due to being retired from the profession and less able to comment on contemporary experiences. The majority (87%) of the sample identified as female. Due to violations of assumptions and statistical power we were unable to include demographic data in all analyses. A decision was made to exclude the four participants who identified as either non-binary, transgender or did not disclose their gender from gender analyses. This was undertaken not with the intention of being non-inclusive but rather to avoid artificially and inappropriately reassigning these individuals to a gender category with which they do not identify. Therefore, the final sample comprised 408 currently practicing veterinary professionals, with 404 respondents included in gender analyses (see Table 1).

**Table 1:** Sociodemographic characteristics of participants (*n* = 408).

	***n***	**%**
**Gender**
Female	355	87
Male	48	11.8
Non-binary	1	0.2
Transgender	1	0.2
Prefer not to disclose	2	0.5
**Occupation**
Veterinarian	241	59.1
Veterinary nurse	156	38.2
Veterinary technician	10	2.5
**Years**' **experience**
1–3 years	108	26.5
4–6 years	84	20.6
7–10 years	62	15.2
11–20 years	75	18.4
More than 20 years	78	19.1
**Type of veterinary practice**
Small animal	315	77.2
Large animal/livestock	16	3.9
Equine	12	2.9
Exotic animal/wildlife	12	2.9
University/research	9	2.2
Other^a^	43	
**Workplace location**
Urban	270	66.2
Rural	106	26
Remote	32	7.8

^a^Breakdown of other veterinary practice types included in Supplementary materials.

### Perceived stigma, barriers to care, fatigue, burnout, and sickness presenteeism

Means and standard deviations are displayed in Table 2 with correlations of continuous variables. Mean scores on both perceived stigma and barriers to care were in the moderate range. Further analysis revealed the barriers perceived to be most frequently impacting access to mental health supports (as indicated by a response of Agree or Strongly agree). These were difficulty getting time off work (58.1%), difficulty scheduling an appointment (57.4%), and the high cost of treatment (52.2%; see Supplementary materials for responses to all subscale items). The majority of participants (88%, *n* = 362) agreed or strongly agreed that fatigue was inevitable in their occupational role. Similarly, 72% (*n* = 296) agreed or strongly agreed that burnout is considered a normal part of the job and 73% (*n* = 299) reported feeling pressure to go into work despite being sick.

**Table 2:** Descriptive statistics and correlations of continuous variables.

		***M* (SD)^+^**	***M* (SD)^∧^**	**Comparison group^e^ *M* (SD)^∧^**	**1**.	**2**.	**3**.	**4**.	**5**.	**6**.
1.	Perceived stigma	15.26 (5.91)	2.56 (0.98)	3.08 (1.04)		0.30**	0.14*	0.22**	0.20**	0.25**
2.	Barriers to care	13.91 (4.15)	2.78 (0.83)	2.56 (0.84)			0.22**	0.34**	0.36**	0.41**
3.	Fatigue^a^	4.25 (0.84)						0.48**	0.29**	0.71**
4.	Burnout^b^	3.78 (1.06)							0.37**	0.80**
5.	Sickness presenteeism^c^	3.88 (1.19)								0.77**
6.	Normalized psycho/pathology^d^	11.91 (2.39)								

^+^Summed scores range from 6 to 30 (perceived stigma) and 5 to 25 (barriers to care).

^∧^Scores range from 1 (lower perceived stigma/barriers) to 5 (higher perceived stigma/barriers).

^**^p < 0.001, *p < 0.01, ^a^Fatigue is inevitable in my role, ^b^Experiencing burnout is considered a normal part of the job, ^c^I feel pressured to go to work even when I am sick, ^d^Summed score of ^a, b^, and ^c^, ^e^Military personnel surveyed 3 months post-deployment (25).

### Effect of gender

No significant effect of gender was found for perceived stigma. Gender was found to have a significant moderate effect on barriers to care, F(1,398) = 27.69, *p* < 0.0005, F(4,399) = 9.66, *p* < 0.0005, η2 = 0.01= 0.06, with females reporting a greater perception of barriers (*M* = 14.33, SD = 3.98) than males (*M* = 11.04, SD = 4.33). Females also reported significantly higher rates of normalized psycho/pathology (*M* = 12.10, SD = 2.26) than males (*M* = 10.72, SD = 2.64; F[1, 401] = 14.67, *p* ≤ 0.001).

### Effect of years' experience

**Figure 1:**
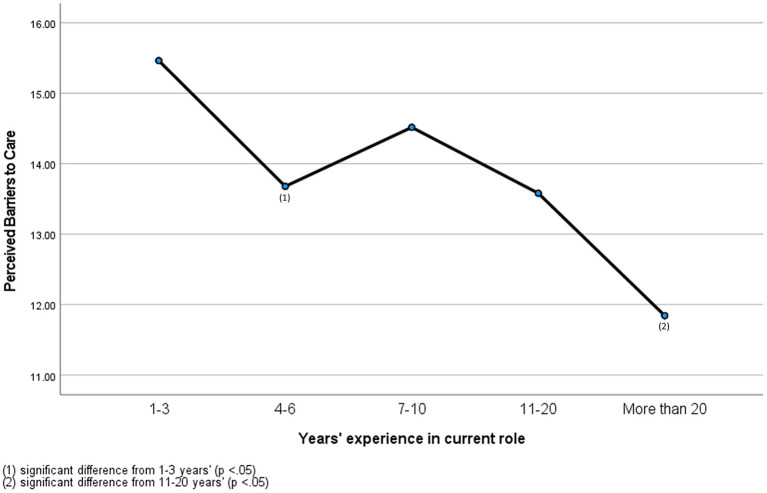
Perceived barriers of veterinary professionals with different years' experience.

Years' experience was not found to significantly predict perceived stigma. There was a significant small effect of years' experience found on reported barriers F(4,399) = 9.66, *p* < 0.0005, η2 = 0.01. Experiences with barriers to care were highest for veterinary professionals with 1–3 years' experience (*M* = 15.46, SD = 3.71), followed by 7–10 years' (*M* = 14.51, SD = 4.24), 4-6 years' (*M* = 13.67, SD = 4.00), 11–20 years' (*M* = 13.64, SD = 3.82) and over 20 years' (*M* = 11.84, SD = 4.28). *Post-hoc* comparisons revealed professionals working in the industry for 1–3 years had significantly higher perceptions of barriers to care than their peers with 4–6 years' experience (*p* = 0.02). Non-statistically significant flucations in barriers to care perception followed professionals from 4–6 years' to 11–20 years' experience. Participants with over 20 years' experience in the industry had significantly lower perceptions of barriers to care than their peers with 11-20 years' experience (*p* = 0.04; see Figure 1).

**Figure 2:**
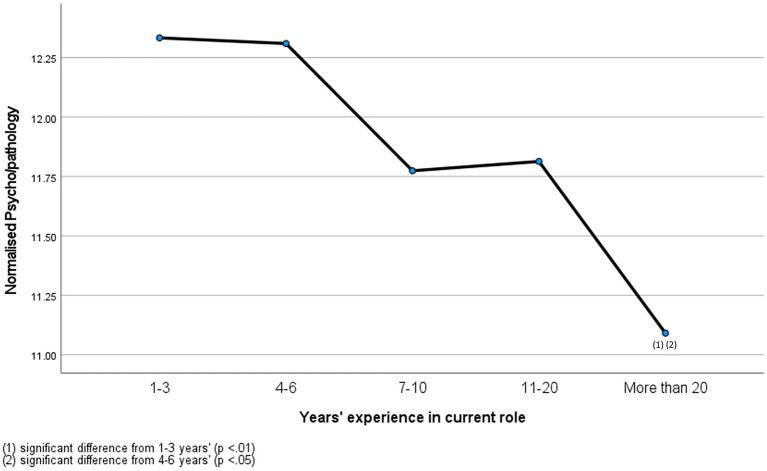
Normalized psychopathology of veterinary professionals with different years' experience.

A significant small effect of years' experience was also found on normalized psycho/pathology F(4,402) = 3.88, p.004, η2 = 0.04 (Figure 2). Experiences with normalized psycho/pathology were highest for veterinary professionals with 1–3 years' experience (*M* = 12.33, SD = 1.88), followed by 4–6 years' (*M* = 12.31, SD = 2.31), 11–20 years' (*M* = 11.81, SD = 2.52), 7–10 years' (*M* = 11.77, SD = 2.54) and over 20 years' experience (*M* = 11.09, SD = 2.66). *Post-hoc* comparison analyses revealed significant differences between professionals with 1–3 years' experience and over 20 years' experience (*p* = 0.004), and 4–6 years' and over 20 years' experience (*p* = 0.01).

### Effect of location

No significant effect of practice location was found on any of the outcome variables.

## Discussion

The present study conducted a preliminary examination of perceived stigma, practical barriers, and normalized psycho/pathology in the Australian and New Zealand veterinary industries. The study sample included a combination of veterinarians, veterinary nurses, and veterinary technicians from an array of practice types and location, with varying years' experience working in animal care.

The moderate mean score for perceived stigma in the study sample is inconsistent with past literature, which maintains stigma is a significant and concerning issue inundating the veterinary industry (8). For example, veterinarians were found to hold more stigma toward mental ill-health than the general US population and less likely to agree that people are caring toward individuals experiencing mental illness (11). This could be due in part to mental health in the industry being a topic of recent discussion in mainstream media. For example, the recent increase in discussion of suicide and mental ill-health rates of veterinary professionals in the media may have encouraged staff to be forthcoming and disclose when they are struggling psychologically. It should be highlighted that the standard deviation for perceived stigma scores in the present study was 5.91 (*M* = 15.26), indicating a diverse range of individual experiences with stigma in the workplace. Due to heterogeneity in the use of the Perceived Stigma and Barriers to Care for Psychological Treatment Scale (e.g., addition and removal of items, alteration of response format), meaningful comparisons with other populations are limited. One sample of military personnel surveyed at 3 months post-deployment observed comparable mean scores of 3.08 (SD = 1.04) for the stigma subscale [mean scores were 2.56 (SD = 0.98) in the present study] (25). The present study did not observe any effects of gender or years' experience, suggesting there may be other predictors involved that further research could explore and identify. Alternative factors influencing stigma that have been observed in other professional groups and populations include mental health literacy, political orientation, and knowing someone experiencing mental illness (26–28). An absence of gender effect should be considered with a reminder that this analysis did not include the data of non-binary and non-disclosing participants and is therefore not generalizable beyond the male/female binary.

Scores indicating perceived barriers to care (e.g., difficulty getting time off work, high cost of treatment) were also in the moderate range but demonstrated effects according to gender and years' experience. Female professionals reported a higher perception of barriers to care in the sample. Despite being a largely female-dominated industry with females comprising around 80% of graduates (29), traditional gendered workforce barriers, such as limited flexibility for working mothers and pay inequality remain ubiquitous in the veterinary industry (30, 31). These are likely functioning to obstruct access to mental health supports for female professionals who are balancing caregiving and work on lower wages than their male counterparts. Further investigation is needed to confirm this effect of gender due to the small proportion of male respondents in the study sample. Similar mean scores to the present study [*M* = 2.78 (SD = 0.83)] were observed in the comparison sample of military personnel [*M* = 2.56 (SD = 0.84)] (25). Early career veterinary professionals (ECVPs) with 1–3 years' experience also reported higher perceptions of barriers to care than their more experienced colleagues. This is consistent with other findings in human healthcare (19) and may be reflective of ECVPs not yet having the knowledge of available supports or practical issues such as time and finances impeding access to such supports. The barriers most reported by participants (difficulty getting time off work, difficulty scheduling an appointment, cost of treatment) are particularly relevant to ECVPs. High levels of student debt and poor remuneration have been recognized as negatively impacting new veterinary graduates (31). Starting remuneration for early career veterinary technicians have been reported as low as AUD$18 per hour in New Zealand and AUD$34 000 per annum in Australia (32). It is therefore likely that ECVPs cannot afford to take time off work to access treatment, nor the cost of treatment itself. The linear decline in perceptions of barriers to mental health help-seeking demonstrated in the current sample may illustrate professionals successfully navigating their way into more senior roles as they acquire more experience in the industry. In addition to higher remuneration, senior positions would provide professionals with more security in their roles and increased locus of control over potential barriers to help-seeking (such as working hours) which would enable professionals to access supports more easily when they are needed.

The notion that mental ill-health symptoms are being normalized in the veterinary profession was supported in the present study. Most respondents endorsed the inevitability of fatigue and burnout in their occupational role and acknowledged feeling pressured to work while they are sick. These responses indicate a central barrier to help-seeking is not fear of negative appraisal (i.e., stigma toward mental ill-health), but a culture of apathy to the full extent of harm posed by these symptoms. Failure to acknowledge fatigue and burnout as symptoms of mental ill-health and instead internalizing them as inevitable increases risk for further deterioration of wellbeing as symptoms are left untreated. Professionals may be dissuaded from accessing needed support if it is perceived as unnecessary or excessive and instead engage in sickness presenteeism.

Sickness presenteeism has been associated with exacerbation of illness and prolonged future absenteeism for employees, compromised patient care and economic consequences for organizations (9, 10). Highly specialized and demanding industries such as healthcare have been identified as particularly vulnerable for staff engaging in this behavior. This has been partly attributed to the sense of moral obligation felt toward professional colleagues and those they are helping (33). In a sample of veterinarians in Australia, 80% of those who endorsed their attendance at work despite displaying symptoms consistent with influenza cited not wanting to burden their colleagues as a reason (9). The authors also argued that the workforce shortage overwhelming the industry means finding staff to cover unplanned absences is often not possible.

**Figure 3:**
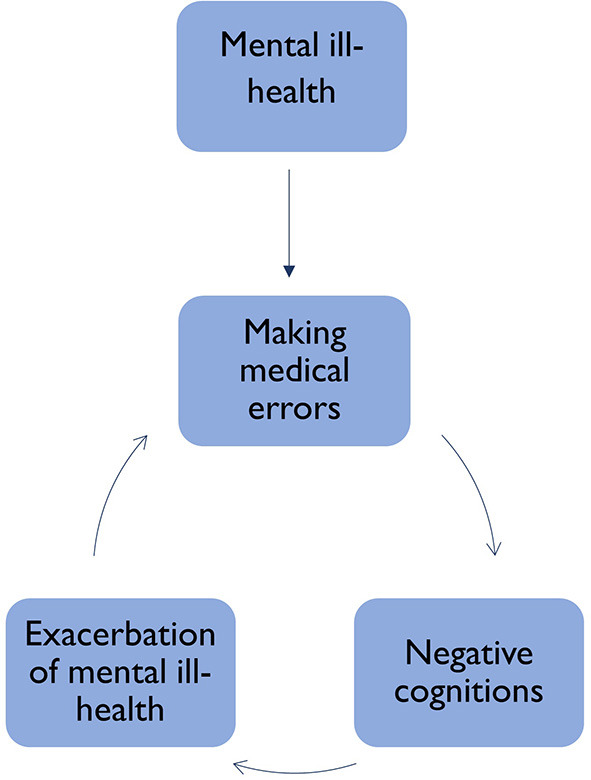
Cycle of sickness presenteeism and mental ill health in the veterinary industry.

It is likely the extent of sickness presenteeism in veterinary professionals experiencing psychological symptoms of ill-health is much higher than physical given the high reported rates of mental ill-health in the industry. The traits commonly observed in veterinary students and professionals, such as perfectionism and concern of how they are perceived by others (22, 34) are also likely to contribute to reluctance in help-seeking or requesting time off due to fears of being perceived as not keeping up with peers. This culture of sickness presenteeism carries an additional risk to the health of patients, with evidence linking burnout to making medical errors (35, 36). Burnout, mental ill-health and threatened patient safety have been suggested to operate in a “vicious cycle” in human healthcare professionals [(33) p. 17]. If a similar cycle were to operate in animal healthcare, this could see professionals who present sick to work with mental and physical ill-health making professional errors which would likely lead to negative self-talk and further exacerbate their ill-health (Figure 3).

### Recommendations for practice and research

Supporting the mental health and wellbeing of the veterinary workforce through both research and policy implementation is imperative to ensuring the sustainability of the profession. The findings of the present study suggest the dominant narrative of stigma toward mental ill-health in the animal care industry is not the sole barrier to help-seeking behaviors in professionals working in Australia and New Zealand, and that practical barriers and cultural norms also play a role. The organizational cultural issue of normalized psycho/pathology would be best addressed with preventative measures at the practice/organizational level. Changes to work, health and safety policy and regulation of work-related psychosocial hazards in Australia are occurring currently. A National code of practice around the protection of employee mental health (37) puts greater onus on employers and industries to eliminate or mitigate work-related risks to mental health (such as excessive job demands) and prevent psychological injury. A recent WorkSafe New Zealand report (38) details similar policy developments are ongoing in New Zealand, emphasizing the importance of identification and reduction of psychosocial workplace risks. These changes to the regulatory environment are designed to encourage better practice and further enable detection and prosecution of poor practice. At the education and training level the importance of prioritizing mental health can be stressed to veterinary students and the normalization of symptoms of mental ill-health and notion of sickness presenteeism being synonymous with work ethic can be debunked. Additional care could be taken to identify those veterinary students notably high in perfectionism, as this has been correlated with neuroticism (39) and therefore place these individuals at greater vulnerability to experiencing occupational distress when entering the workforce. Education highlighting the negative outcomes that unrealistic expectations may elicit is recommended, alongside increased awareness of the reality and frequency of imperfect experiences and outcomes (39).

Concurrently, additional barriers identified in the present study such as time and financial constraints can be considered by organizations when developing workplace policy to support employee wellbeing. Allocation of time during work hours and provision of financial support for relevant resources and evidence-informed training programs will assist those impeded by time or the cost of psychotherapeutic treatments. Encouraging professionals to take time off when they are not well may also prevent the onset of more serious conditions and therefore benefit employees, employers, clients, and patients. However, this may not be feasible until staffing shortages in the industry (31) are rectified. The challenges associated with recruitment and retention within the industry in both Australia^1^ and New Zealand^2^ signal a more comprehensive approach is needed that systematically embeds skills in identifying and addressing mental health needs at a personal, professional, and organizational level throughout the career trajectory from training to continuing professional development. Employee wellbeing in animal care should be framed as an organizational investment, rather than a time and financial burden.

When considering the above recommendations particular focus should be placed on vulnerable groups such as early career veterinary professionals who may be at elevated risk for encountering barriers to mental health help-seeking. Veterinary students and recent graduates reported high rates of mental ill-health including of depression, anxiety and psychological distress (40, 41). Symptoms of depression, stress and burnout have all been observed as highest in the initial years following graduation (3). The transition from university to practicing in the workplace has been highlighted as a particularly difficult transition for graduates and characterized by a chronic absence of support (42). In a sample of recent veterinary graduates from New Zealand, 44% had left their first job and cited lack of support as one of the main contributors. Therefore, it would be useful to direct efforts to increasing perceptible support and targeting other stressors relevant to ECVPs to foster wellbeing and reduce the need for mental health help-seeking. This may also be more practicable until issues such as staff shortages are addressed. The additional benefits of this approach extend beyond the individual to the workplace, industry and service users as increase wellbeing is associated with staff retention and improved work performance (43). Although the results of the present study do not highlight professionals located in rural and remote areas as more susceptible to experiencing barriers to mental health help-seeking, the shortage of veterinary professionals in these areas is regularly reported and it is more than likely this demographic experiences unique obstacles to providing care that are beyond the scope of this paper. Additionally, these results may have been skewed by only one third of respondents working in rural or remote areas.

However, more research needs to be conducted alongside these practical measures to attain the required knowledge to effectively support this population. The research base examining healthcare professionals tends to be dominated by human healthcare, with substantially less focus on veterinary professionals in comparison. The results of this preliminary investigation indicate this attention needs to extend to include veterinary care, highlighting an important area for future research to determine barriers to mental health help-seeking more conclusively. A compounding shortcoming is the largely heterogeneous methodology that exists in the empirical research literature. Despite well intentioned efforts, this diversity is considered to be preventing meaningful synthesis of data and ultimately hindering progress (44). Furthermore, there is currently no standardized measure to examine normalized psycho/pathology leading the authors of the present study to develop new questionnaire items which further slows the understanding of this population and development of interventions to minimize psychological distress. The development and employment of systematic methods and psychometric instruments should be prioritized to assist with closing the knowledge gap between the experiences of human and non-human healthcare professionals. As highlighted in the use of the Perceived Stigma and Barriers to Care for Psychological Treatment Scale, the research space would also greatly benefit from homogenous employment of existing instruments to allow for accurate comparisons between populations.

### Strengths, limitations and future research directions

Although a response rate could not be calculated, the sample size is a strength of this study. The preliminary research presented provides a solid foundation that lends itself to future study in this area with some notable findings relating to industry culture and barriers to mental health and wellbeing that warrant deeper investigation. As the present study was conducted during the COVID-19 pandemic, the generalizability of results must be considered in context with restrictions imposed on professionals, such as separation from friends and family due to mandated lockdowns and social distancing requirements at work. It is possible these parameters exacerbated feelings of isolation, and thereby perceptions of barriers to mental health care (although studies published pre-pandemic demonstrate long working hours in social isolation has been an ongoing stressor for veterinary professionals; 3). Future research would be beneficial in examining these perceptions over time as public health orders are eased and industries adjust to operating in a pandemic. Employing a longitudinal study design would also be valuable in overcoming the limitations typical to a cross-sectional study (i.e., inability to make causal inference or establish the temporal relation between predictors and outcomes) and determining how perceptions of mental health care and stigma toward psychological ill-health change over time worked in the industry. Finally, voluntary participation may have introduced a bias into the resulting sample population to those more motivated to engage due to the incentive offered or their own experiences of mental ill-health.

## Conclusions

The present study examined perceptions of help-seeking behaviors by veterinary professionals, and the normalization of psycho/pathology in the animal care industry. The results highlight the need for more emphasis on observable and practical support in fostering an industry in which employees are encouraged to be forthcoming with their mental health challenges and seek help when it is required. Additional efforts are required in the research space to advance our understanding of the experiences of this population and progress intervention development.

## Data availability statement

The original contributions presented in the study are included in the article/Supplementary material, further inquiries can be directed to the corresponding author/s.

## Ethics statement

The studies involving human participants were reviewed and approved by Tasmania Social Sciences Human Research Ethics Committee. The patients/participants provided their written informed consent to participate in this study.

## Author contributions

CC conceived the study, conducted the research, and wrote the manuscript. KN assisted with the study design and data analysis. KN, SD, and AM edited the manuscript before submission. All authors contributed to the article and approved the submitted version.

## Conflict of interest

The authors declare that the research was conducted in the absence of any commercial or financial relationships that could be construed as a potential conflict of interest.

## Publisher's note

All claims expressed in this article are solely those of the authors and do not necessarily represent those of their affiliated organizations, or those of the publisher, the editors and the reviewers. Any product that may be evaluated in this article, or claim that may be made by its manufacturer, is not guaranteed or endorsed by the publisher.

## Footnotes

^1^https://www.abc.net.au/news/rural/2022-06-05/vets-eyre-peninsula-under-pressure-national-shortage/101116706.

^2^https://nzva.org.nz/professionals/advocacy-consultation/advocacy/vet-shortage-nz/.

## References

[1] Andela M. Work-related stressors and suicidal ideation: the mediating role of burnout. J Workplace Behav Health. (2021) 36:125–45. 10.1080/15555240.2021.1897605

[2] Schwerdtfeger KA Bahramsoltani M Spangenberg L Hallensleben N Glaesmer H. Depression, suicidal ideation and suicide risk in German veterinarians compared with the general German population. Vet Rec. (2020) 186:e2. 10.1136/vr.105430 32229508

[3] Hatch PH Winefield HR Christie BA Lievaart JJ. Workplace stress, mental health, and burnout of veterinarians in Australia. Aust Vet J. (2011) 89:460–8. 10.1111/j.1751-0813.2011.00833.x 22008127

[4] Connolly CE Norris K Martin A Dawkins S Meehan C. A taxonomy of occupational and organisational stressors and protectors of mental health reported by veterinary professionals in Australasia. Aust Vet J. (2022) 100:367–76. 10.1111/avj.13167 35560212 PMC9544948

[5] Gardner DH Hini D. Work-related stress in the veterinary profession in New Zealand. New Zeal Vet J. (2006) 54:119–24. 10.1080/00480169.2006.36623 16751842

[6] Bartram DJ Baldwin DS. Veterinary surgeons and suicide: a structured review of possible influences on increased risk. Vet Rec. (2010) 166:388–97. 10.1136/vr.b4794 20348468

[7] Dow MQ Chur-Hansen A Hamood W Edwards S. Impact of dealing with bereaved clients on the psychological wellbeing of veterinarians. Aust Vet J. (2019) 97:382–9. 10.1111/avj.12842 31364771

[8] Platt B Hawton K Simkin S Dean R Mellanby RJ. Suicidality in the veterinary profession: interview study of veterinarians with a history of suicidal ideation or behavior. Crisis. (2012) 33:280–9. 10.1027/0227-5910/a000143 22713972

[9] Pasfield K Gottlieb T Tartari E Ward MP Quain A. Sickness presenteeism associated with influenza-like illness in veterinarians working in New South Wales: results of a state-wide survey. Aust Vet J. (2022) 100:243–53. 10.1111/avj.13153 35168290 PMC9304280

[10] Skagen K Collins AM. The consequences of sickness presenteeism on health and wellbeing over time: a systematic review. Soc Sci Med. (2016) 161:169–77. 10.1016/j.socscimed.2016.06.005 27310723

[11] Nett RJ Witte TK Holzbauer SM Elchos BL Campagnolo ER Musgrave KJ. Risk factors for suicide, attitudes toward mental illness, and practice-related stressors among US veterinarians. J Am Vet Med A. (2015) 247:945–55. 10.2460/javma.247.8.945 26421408

[12] Zenner D Burns GA Ruby KL Debowes RM Stoll S. Veterinary students as elite performers: preliminary insights. J Vet Med Educ. (2005) 32:242–8. 10.3138/jvme.32.2.242 16078178

[13] Lokhee S Hogg RC. Depression, stress and self-stigma towards seeking psychological help in veterinary students. Aust Vet J. (2021) 99:309–17. 10.1111/avj.13070 33880748

[14] Britt TW Greene–Shortridge TM Brink S Nguyen QB Rath J Cox AL. Perceived stigma and barriers to care for psychological treatment: implications for reactions to stressors in different contexts. J Soc Clin Psychol. (2008) 27:317–35. 10.1521/jscp.2008.27.4.317

[15] Clough BA March S Leane S Ireland MJ. What prevents doctors from seeking help for stress and burnout? A mixed-methods investigation among metropolitan and regional-based australian doctors. J Clin Psychol. (2019) 75:418–32. 10.1002/jclp.22707 30431644

[16] Animal Medicines Australia. Pets and the Pandemic: A Social Research Snapshot of Pets and People in the COVID-19 Era. Barton, ACT: Animal Medicines Australia (2021).

[17] Bryant L Garnham B Tedmanson D Diamandi S. Tele-social work and mental health in rural and remote communities in Australia. Int Soc Work. (2015) 61:143–55. 10.1177/0020872815606794

[18] St Clair M Murtagh D. Barriers to telehealth uptake in rural, regional, remote australia: what can be done to expand telehealth access in remote areas? Stud Health Technol Inform. (2019) 266:174–82. 10.3233/SHTI190791 31397320

[19] Ramzi NSAM Deady M Petrie K Crawford J Harvey SB. Help-seeking for depression among Australian doctors. Intern Med J. (2021) 51:2069–77. 10.1111/imj.15035 32833296

[20] Ogrodniczuk JS Oliffe JL. Men and depression. Can Fam Physician. (2011) 57:153–5. 21321163 PMC3038800

[21] Gold KJ Andrew LB Goldman EB Schwenk TL. “I would never want to have a mental health diagnosis on my record”: a survey of female physicians on mental health diagnosis, treatment, and reporting. General Hosp Psychiat. (2016) 43:51–7. 10.1016/j.genhosppsych.2016.09.004 27796258

[22] Hancock TS Karaffa KM. “Obligated to Keep Things Under Control”: sociocultural barriers to seeking mental health services among veterinary medical students. J Vet Med Educ. (2021) 49:662–77. 10.3138/jvme-2021-0069 34460356

[23] Winter P Rix A Grant A. Medical student beliefs about disclosure of mental health issues: a qualitative study. J Vet Med Educ. (2017) 44:147–56. 10.3138/jvme.0615-097R 28206830

[24] Hoge CW Castro CA Messer SC McGurk D Cotting DI Koffman RL. Combat duty in Iraq and Afghanistan, mental health problems, and barriers to care. N Engl J Med. (2004) 351:13–22. 10.1056/NEJMoa040603 15229303

[25] Kim P Thomas JL Wilk JE Castro CA Hoge CW. Stigma, barriers to care, and use of mental health services among active duty and national guard soldiers after combat. Psychiatr Serv. (2010) 61:582–8. 10.1176/ps.2010.61.6.582 20513681

[26] DeLuca JS Vaccaro J Seda J Yanos PT. Political attitudes as predictors of the multiple dimensions of mental health stigma. Int J Soc Psychiatry. (2018) 64:459–69. 10.1177/0020764018776335 30051764

[27] Griffiths KM Christensen H Jorm AF. Predictors of depression stigma. BMC Psychiatry. (2008) 8:25. 10.1186/1471-244X-8-25 18423003 PMC2386456

[28] Soomro S Yanos PT. Predictors of Mental Health Stigma among Police Officers: the Role of Trauma and PTSD. J Police Crim Psychol. (2018) 34:175–83. 10.1007/s11896-018-9285-x

[29] Irvine L Vermilya JR. Gender work in a feminized profession. Gender Soc. (2010) 24:56–82. 10.1177/0891243209355978

[30] Treanor L Marlow S. Paws for thought? Analysing how prevailing masculinities constrain career progression for UK women veterinary surgeons. Hum Rel. (2021) 74:105–30. 10.1177/0018726719846554

[31] Australian Veterinary Association. Australian Veterinary Workforce Survey 2018. Sydney, NSW: Australian Veterinary Association (2019).

[32] Gates MC Palleson-Putt P Sawicki RK. Veterinary technology graduates' perceptions of their education and subsequent employment experiences. New Zeal Vet J. (2021) 69:93–103. 10.1080/00480169.2020.1837689 33064629

[33] Kinman G. Sickness presenteeism at work: prevalence, costs and management. Br Med Bull. (2019) 129:69–78. 10.1093/bmb/ldy043 30649219

[34] Crane MF Phillips JK Karin E. Trait perfectionism strengthens the negative effects of moral stressors occurring in veterinary practice. Aust Vet J. (2015) 93:354–60. 10.1111/avj.12366 26412116

[35] Hayes GM LaLonde-Paul DF Perret JL Steele A McConkey M Lane WG. Investigation of burnout syndrome and job-related risk factors in veterinary technicians in specialty teaching hospitals: a multicenter cross-sectional study. J Vet Emerg Crit Care. (2020) 30:18–27. 10.1111/vec.12916 31840933 PMC7003767

[36] O'Connor DB Hall LH Johnson J. Job strain, burnout, wellbeing and patient safety in healthcare professionals. In:Montgomery A van der Doef M Panagopoulou E Leiter MP, editors. Connecting Healthcare Worker Well-Being, Patient Safety and Organisational Change: The Triple Challenge. Cham: Springer International Publishing (2020). p. 11–23.

[37] Safe Work Australia. Codes of Practice. Canberra, ACT: Safe Work Australia (2022).

[38] Lovelock K. Psychosocial Hazards in Work Environments and Effective Approaches for Managing Them. Wellington: WorkSafe New Zealand (2019).

[39] Holden CL. Characteristics of veterinary students: perfectionism, personality factors, and resilience. J Vet Med Educ. (2020) 47:488–96. 10.3138/jvme.0918-111r 32412364

[40] Reisbig AM Danielson JA Wu TF Hafen M Jr Krienert A Girard D. A study of depression and anxiety, general health, and academic performance in three cohorts of veterinary medical students across the first three semesters of veterinary school. J Vet Med Educ. (2012) 39:341–58. 10.3138/jvme.0712-065R 23187027

[41] Yang HH Ward MP Fawcett A DVM. students report higher psychological distress than the Australian public, medical students, junior medical officers and practicing veterinarians. Aust Vet J. (2019) 97:373–81. 10.1111/avj.12845 31310017

[42] Favier RP Ten Cate O Duijn C Bok HGJ. Bridging the gap between undergraduate veterinary training and veterinary practice with entrustable professional activities. J Vet Med Educ. (2021) 48:136–8. 10.3138/jvme.2019-0051 32149590

[43] Bryson A Forth J Stokes L. Does employees' subjective well-being affect workplace performance? Hum Rel. (2017) 70:1017–37. 10.1177/0018726717693073

[44] Scotney RL McLaughlin D Keates HL A. systematic review of the effects of euthanasia and occupational stress in personnel working with animals in animal shelters, veterinary clinics, and biomedical research facilities. J Am Vet Med A. (2015) 247:1121–30. 10.2460/javma.247.10.1121 26517615

