# Supplementary material for: Barriers to mental health help-seeking in veterinary professionals working in Australia and New Zealand: A preliminary cross-sectional analysis
Source: Front Vet Sci. 2022 Nov 4;9:1051571. doi: 10.3389/fvets.2022.1051571 (PMC9671929; doi:10.3389/fvets.2022.1051571)
Supplement: Supplementary file 1 [file Data_Sheet_1.docx]

Supplementary Material

# Veterinary practice type

| Type of veterinary practice | *n* | % |
| --- | --- | --- |
| Small animal | 315 | 77.2 |
| Large animal/livestock | 16 | 3.9 |
| Equine | 12 | 2.9 |
| Exotic animal/wildlife | 12 | 2.9 |
| University/research | 9 | 2.2 |
| Other | 43 | 10.5 |

## ‘Other’ veterinary practice types (reported via free text option)

| Type of veterinary practice | *n* | % |
| --- | --- | --- |
| Animal shelter | 2 | 0.4 |
| Aquaculture | 1 | 0.2 |
| Emergency | 4 | 0.8 |
| Mixed | 20 | 4.8 |
| Government | 1 | 0.2 |
| Livestock consultancy | 1 | 0.2 |
| Pathology | 1 | 0.2 |
| Pharmaceutical industry | 1 | 0.2 |
| Specialist | 1 | 0.2 |
| Veterinary teaching hospital | 2 | 0.4 |

# Barriers to mental health help-seeking data

| Barrier | Strongly disagree *n* (%) | Disagree *n* (%) | Neither agree nor disagree *n* (%) | Agree *n* (%) | Strongly agree *n* (%) |
| --- | --- | --- | --- | --- | --- |
| I don’t know where to get help | 145 (35.5) | 98 (24) | 63 (15.4) | 72 (17.6) | 27 (6.6) |
| I don’t have adequate transportation | 341 (83.6) | 40 (9.8) | 14 (3.4) | 7 (1.7) | 4 (1.0) |
| It is difficult to schedule an appointment | 57 (14) | 47 (11.5) | 69 (16.9) | 113 (27.7) | 121 (29.7) |
| There would be difficulty getting time off work/school for treatment | 67 (16.4) | 49 (12) | 55 (13.5) | 120 (29.4) | 117 (28.7) |
| Getting treatment costs too much money | 55 (13.5) | 50 (12.3) | 90 (22.1) | 107 (26.2) | 106 (26) |
